# Recurrence and prognostic factors in patients with aggressive fibromatosis. The role of radical surgery and its limitations

**DOI:** 10.1186/1477-7819-10-184

**Published:** 2012-09-10

**Authors:** Emilio Bertani, Alessandro Testori, Antonio Chiappa, Pasquale Misitano, Roberto Biffi, Giuseppe Viale, Giovanni Mazzarol, Tommaso De Pas, Edoardo Botteri, Gianmarco Contino, Francesco Verrecchia, Barbara Bazolli, Bruno Andreoni

**Affiliations:** 1 Division of General and Laparoscopic Surgery, European Institute of Oncology, Via G. Ripamonti, 435, 20141, Milan, Italy; 2 Division of Melanoma and Muscle-Cutaneous Sarcomas, European Institute of Oncology, Via G. Ripamonti, 435, 20141, Milan, Italy; 3 University of Milan, Milan, Italy; 4 Division of Abdomino-Pelvic Surgery, European Institute of Oncology, Via G. Ripamonti, 435, 20141, Milan, Italy; 5 Division of Pathology, European Institute of Oncology, Via G. Ripamonti, 435, 20141, Milan, Italy; 6 Division of Medical Oncology, European Institute of Oncology, Via G. Ripamonti, 435, 20141, Milan, Italy; 7 Division of Epidemiology and Biostatistics, European Institute of Oncology, Via G. Ripamonti, 435, 20141, Milan, Italy

**Keywords:** Aggressive fibromatosis, Desmoid tumors, Surgery, Frozen sections, Local recurrence, Risk factors

## Abstract

**Background:**

Surgery is still the standard treatment for aggressive fibromatosis (AF); however, local control remains a significant problem and the impact of R0 surgery on cumulative recurrence (CR) is objective of contradictory reports.

**Methods:**

This is a single-institution study of 62 consecutive patients affected by extra-abdominal and intra-abdominal AF who received macroscopically radical surgery within a time period of 15 years.

**Results:**

Definitive pathology examination confirmed an R0 situation in 49 patients and an R1 in 13 patients. Five-year CR for patients who underwent R0 vs R1 surgery was 7.1% vs 46.4% (*P* = 0.04) and for limbs vs other localizations 33.3% vs 9.9% (*P* = 0.02) respectively. In 17 patients who had intraoperative frozen section (IFS) margin evaluation R0 surgery was more common (17 of 17 vs 32 of 45, *P* = 0.01) and CR lower (five-year CR 0% vs 19.1%, respectively, *P* = 0.04). However, in multivariate analysis only limb localization showed a negative impact on CR (HR: 1.708, 95% CI 1.03 to 2.84, *P* = 0.04).

**Conclusions:**

IFS evaluation could help the surgeon to achieve R0 surgery in AF. Non-surgical treatment, including watchful follow-up, could be indicated for patients with limb AF localization, because of their high risk of recurrence even after R0 surgery.

## Background

Among soft tissue neoplasms, desmoids tumors, also called aggressive fibromatosis, account for less than 3% (0.03 of all tumors). These neoplasms do not have metastatic potential but tend to locally infiltrate the musculo-aponeurotic structures [1]. Local control of aggressive fibromatosis (AF) remains a significant problem, with an average recurrence rate of 24 to 77% no matter what therapeutic modality is used [2]. Surgery is the primary therapy for extra-abdominal and abdominal wall desmoid tumors; however, the identification of informative prognostic factors, such as margins (R0 surgery), localization, diameter and so on is still controversial. Of particular importance for the surgeon is the prognostic significance of R0 surgery in planning the width of resection, especially when the surgical site is challenging and surgery has the risk of short- and long-term postoperative complications. In addition, alternative approaches, such as radiotherapy [3,4], COX-2 inhibitors [5], anti-estrogens, interferon alpha, vitamin C [6], cytotoxic chemotherapy [7,8] and imatinib [9], have shown various degrees of efficacy. Observation alone is increasingly recommended for static lesions, given the morbidity associated with surgical resection and frequent disease recurrence [10,11]. Therefore, a consensus over the standard of care is limited and weakened by heterogeneous treatments and lack of large studies. In this context, the aim of the present study was to analyze the role and the limitations of radical surgery and the impact of the other risk factors for AF recurrence in a consecutive surgical series of patients homogeneously treated at a single institute.

## Methods

From 1994 to 2010, 73 patients affected by AF were observed at the European Institute of Oncology and their records were extracted from the institute’s tumor registry, a prospective desmoid tumor database containing 65 data fields. Eleven patients had a histologically confirmed diagnosis of AF in the resected specimen or pathology review in our institute. One patient was affected by familial adenomatous polyposis coli (FAP). Sixty-three patients underwent surgery (86.3%), while the remaining 10 patients were judged unresectable. Among this latter group, two cases underwent low-dose chemotherapy, three were given COX-2 inhibitors with or without tamoxifen, while four patients were put under observation. Of the 63 surgically treated patients, 62 (98%) received macroscopically radical surgery (R0 or R1 according to AJCC) [12] and form the body of the analysis in the present study. Written informed consent was obtained from the patients for publication of this report and any accompanying images.

### Pre-treatment work-up

Twenty-eight patients were studied by magnetic resonance imaging (MRI), and 34 by computed tomography (CT) scan. Ultrasound (US) examination was the only diagnostic tool employed before treatment for 10 patients. US-guided core biopsy (Gallini- Mantova, Italy ) was performed in 44 cases (71%), which was diagnostic for AF in 37 patients and nondiagnostic in the remaining 7.

### Surgical technique

Surgical principles that aided resection comprised a wide excision of the mass, which involved the removal of all gross disease together with a normal tissue rim of at least 1 cm whenever possible [13]. For this purpose, intraoperative frozen section (IFS) margin evaluation was employed in those cases that the operating surgeon deemed at risk of margin involvement. All operations were performed under antibiotic cover with third generation cephalosporin, which was continued until the suction drains were removed. Low molecular weight heparins were administered starting from the first preoperative day and were continued for one month following surgery to prevent thromboembolic disease.

### Pathology examination

Both the surgical specimen and margin biopsy where necessary were oriented by the surgeon and inked by the pathologist. In cases of IFS diagnosis, the pathologist examined all the margins if the neoplasm was ill-defined or the closest margin to neoplasia in the case of a clearly recognizable mass. Biopsy specimens were analyzed in their entirety. If neoplastic cells were detected on the inked margin examined, further surgery was performed until negative microscopic margins were achieved [14]. A histological diagnosis of aggressive fibromatosis was obtained in all operated cases. Whenever deemed necessary, appropriate immunohistology was performed as an adjunct to the morphological evaluation of the neoplasia. Tumor characteristics studied included site and size: the size was obtained by measuring the largest dimension of the tumor in the surgical specimen and by MRI or CT scan for non-operated patients.

### Follow-up and recurrence

Follow-up was by outpatient clinical appointments, with clinical and radiological assessment as indicated. Recurrence was diagnosed radiologically and confirmed by percutaneous biopsy when appropriate. No patient was lost to follow-up.

### Statistical analysis

The Chi-square test and the Fisher exact test were used, as appropriate, to compare distributions of categorical variables. The crude cumulative incidence of tumor recurrences was computed in a competing risk framework, with deaths without recurrence treated as competing events. Cumulative incidences were compared across subgroups by means of the Gray test. A multivariable Cox regression model was used to test the independent prognostic value of the variables which showed significant results in the univariate analysis. Adjusted hazard ratios (HR) with 95% confidence intervals (CIs) were reported. All analyses were carried out with the SAS software (SAS Institute, Cary, NC, USA) and the R (http://cran.r-project.org/) software. All the reported *P*-values were two-sided.

## Results

There were 43 females and 19 males (median age 36 years, range 16 to 76 years) affected by primary (52) or recurrent (10) disease. In order to optimize surgical outcome (at least 1 cm of free margin whenever possible) intraoperative frozen section (IFS) evaluation was employed in 17 cases. In six of these cases it was performed twice because the first IFS examination reported a microscopically involved margin. Definitive pathology examination confirmed an R0 situation in 49 patients and R1 in 13 patients. No cases of discordance between the last intraoperative and the final margin evaluation (R0 vs R1) were registered among the 17 patients who had IFS evaluation (Table 1). The probability of receiving IFS evaluation was not correlated to the tumor size (3 of 12 vs 14 of 50 for <10 cm vs ≥10 cm respectively, *P* = 0.83). R1 patients were more likely to receive postoperative radiotherapy (Table 1).

**Table 1:** Factors associated with the achievement of R0 surgery for the 62 radically resected patients

**Variable**	**Classification**	**R0 N = 49**	**R1 N = 13**	***P*-value**
**Age**	≤36	24 (77.4)	7 (22.6)	0.76
	>36	25 (80.7)	6 (19.4)	
**Gender**	M	15 (78.9)	4 (21.1)	1.00
	F	34 (79.1)	9 (20.9)	
**Recurrence at presentation**	Yes	8 (80.0)	2 (20.0)	1.00
	No	41 (78.8)	11 (21.2)	
**Site of tumor**	Trunk	33 (78.6)	9 (21.4)	0.35
	Head and neck	4 (57.1)	3 (42.9)	
	Limbs	6 (85.7)	1 (14.3)	
	Mesentery	6 (100)	0 (0.0)	
**Limb localization**	Yes	6 (85.7)	1 (14.3)	1.00
	No	43 (78.2)	12 (21.8)	
**Tumor maximum diameter**	<10 cm	40 (80.0)	10 (20.0)	0.70
	≥10 cm	9 (75.0)	3 (25.0)	
IFS^*^	No	32 (71.1)	13 (28.9)	0.01
	Yes	17 (100.0)	0 (0.0)	
**Postoperative RT**^**§**^	No	46 (83.6)	9 (16.4)	0.03
	Yes	3 (42.9)	4 (57.1)	

^*^IFS, intraoperative frozen sections; ^§^RT, radiotherapy.

### Follow-up and survival

After a median follow-up time of 66 months (range 2 to 175) two deaths related to the disease were registered. These deaths were not caused by recurrence of the disease, but by long-term complications of treatment. In one case, a 30 year-old male patient affected by a huge intra-abdominal recurrent desmoid, who underwent radical surgery at our institute, died after 14 months becaus of rejection after small bowel transplantation. The other case was a 54 year-old male affected by a cervico-thoracic desmoid, who underwent a huge demolition of the thoracic wall and pulmonary resection, followed by radiotherapy as a result of the pathology findings of R1 surgery, and developed a pulmonary mycetoma that was resected 32 months after the primary surgery. He died from subsequent complications four months thereafter. Three other patients died during follow-up due to causes other than fibromatosis (one for cardiovascular event, the other two for advanced colorectal cancer). For these reasons, actuarial five-year overall survival was 95% in our series.

### Risk factors affecting cumulative recurrence (CR)

Seven patients developed recurrence during follow-up that arose within the surgical field in four cases, and within the same anatomical region, but distant from the surgical field, in the remaining three patients. Of these seven patients, three underwent repeated surgery that was demonstrated to be R0 in all three cases. At this time all three patients are alive and disease-free. None of the patients who had IFS examination developed recurrence during follow-up.

**Figure 1:**
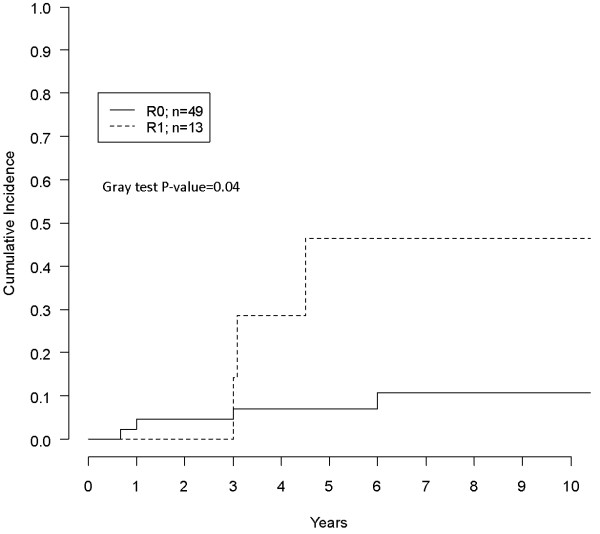
Impact of R0 vs R1 surgery on CR for the 62 patients.

**Figure 2:**
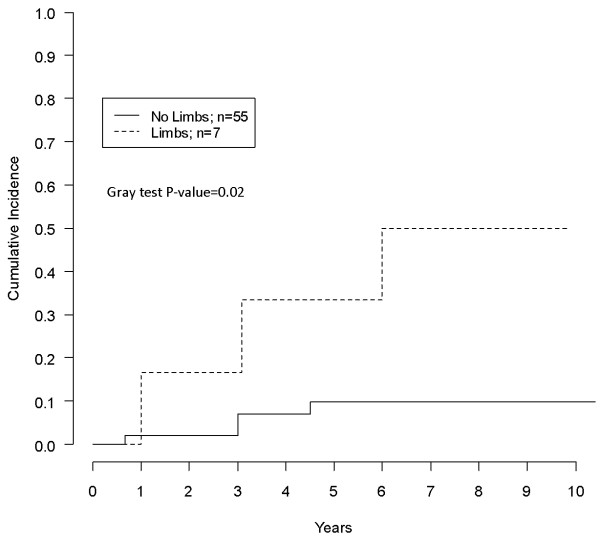
Impact of tumor localization (limbs vs no limbs).

**Figure 3:**
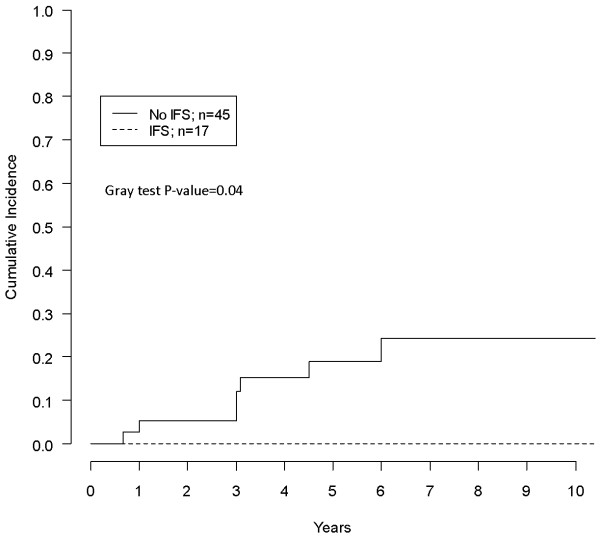
Impact of performing IFS on CR.

Actuarial CR was 8%, 13%, 16% at three to five and eight years respectively. Univariate analysis of possible factors affecting CR is reported in Table 2. R1 surgery, (Figure 1), tumor maximum diameter ≥10 cm and limb localization (vs any other) (Figure 2) were associated with significantly higher CR rates. Patients who had IFS margin evaluation showed a significantly lower CR (no recurrences in that group) in comparison with patients who did not have such an assessment (Figure 3). In multivariate analysis only limb localization was significantly associated with higher CR (Table 3).

**Table 2:** Factors affecting CR rate for the 62 patients who underwent macroscopically radical tumor resection

**Variable**	**Classification**	**At risk**	**Recurrences (% 5-year cumulative incidence)**	***P*-value**
**All patients**		62	7 (12.8)	
**Age**	≤36	31	5 (19.2)	0.30
	>36	31	2 (5.0)	
**Gender**	M	19	2 (13.1)	0.74
	F	43	5 (12.2)	
**Recurrence at**				
**presentation**	Yes	10	3 (10.6)	0.17
	No	52	4 (20.0)	
**Site of tumor**	Trunk	42	1 (7.8)	0.09
	Head and neck	7	1 (16.7)	
	Limbs	7	3 (33.3)	
	Mesentery	6	2 (16.7)	
**Limb localization**	Yes	7	3 (33.3)	0.02
	No	55	4 (9.9)	
**Residual tumor**	R0	49	4 (7.1)	0.04
	R1	13	3 (46.4)	
**Tumor maximum diameter**	<10 cm	50	4 (8.1)	0.05
	≥10 cm	12	3 (31.8)	
IFS^*^	No	45	7 (19.1)	0.04
	Yes	17	0 (0.0)	

^*^IFS, intraoperative frozen sections.

**Table 3:** Multivariate analysis of factors affecting CR

	**HR**	**95 % CI**	***P*-value**
Limb localization^*^	1.71	1.03 to 2.84	0.04
**R1 surgery**^**§**^	2.94	0.47 to 18.20	0.25
**Tumor size (≥10 cm)**	2.68	0.43 to 16.67	0.29

Abbreviation: HR, hazard ratio.

^*^Limbs vs all other localizations.

^§^R1 vs R0 surgery.

## Discussion

Despite the rarity of this disease, several reports exist on prognostic factors for recurrence after resection of AF [15-24]. However, to minimize recurrence, clear indications on how to perform surgery and analyze AF specimens are lacking. In the current single-center study, R1 surgery, tumor diameter (≥10 cm) and limb localization were the most significant prognostic factors associated with higher CR in a consecutive series of desmoid tumors. The achievement of a microscopically negative resection margin assessed by IFS was an accurate predictor of R0 resection and lower CR, where none of the 17 negative IFS specimens was demonstrated to have microscopic infiltration of the margin at definitive pathology stain and none of the patients receiving IFS developed recurrence. This finding is limited by the lack of randomization and by the fact that IFS was employed in only 27% of patients from our series, so that we do not have sufficient data supporting a further reduction of R1 cases with increased use of IFS. Strict cooperation between the team of doctors involved in the management of this disease is of particular importance in achieving successful R0 surgery. The anatomical surgery should be accurately evaluated for a more complete radicality, and the margin at risk of involvement clearly indicated by the surgeon to the pathologist. In addition, pathologists should be fully aware of the characteristics of the disease that they are dealing with, which is often infiltrating, and of any previous treatment, such as radiotherapy or surgery, in order to better interpret any findings of fibrosis, inflammatory cells or cellular atypia in particular at the time when a correct margin definition is needed.

The impact of a negative surgical margin after surgery for desmoid tumors has been widely debated in the literature. In a recently published French study [15], the largest series of sporadic desmoids, progression-free survival curves were not significantly different in terms of microscopic assessment of surgical resection quality (R0 vs R1), although R2 resections showed a significantly poorer prognosis. This French study, which retrospectively entered into a database the data of 426 patients from 24 participating centers starting from 1965, showed a five-year progression-free survival rate of 62.5% for R0 resections, which compares unfavorably with our figure of 92.9%. This difference is difficult to explain, but if we consider that RFS rates for R1 cases are quite similar (60.5% vs 53.6%), it is arguable that in a large multicentric study enrolling very early cases, a problem may be the lack of standardization mainly for pathological assessment of margins (for example, definition of “R0” and “R1” resections). In the study conducted by the Italian National Tumour Institute (INT) [16], the presence of microscopic disease in one surgical margin assumed prognostic relevance in patients with an already recurrent disease but not in patients with primary lesions. Indeed, data from Memorial Sloan-Kettering Cancer Centre (MSKCC) [17] , Mirabell *et al.*[18], Reitamo *et al.*[19] made the same observations. Conversely, and in line with our data, other authors [20-22] identified a positive resection margin as the most important independent predictive factor of local recurrence.

We identify the subgroup of patients affected by AF limb localizations to be at risk for recurrence even after radical surgery. This finding is similar to that of several other reports [15,16,21,22]. In the study carried out by the University of Texas [23] (UTMDACC) authors compared two consecutive series of desmoids tumors investigating differences of approach and outcome between an earlier group of patients treated from 1965 to 1994 and a recent group treated from 1995 to 2005. Interestingly, in the most recent series only extremity site and age ≤30 were prognostic factors for recurrence. Although in that group patients with positive margins were generally more likely to undergo adjuvant therapy (53% vs 23%), the interseries comparison highlighted significant improvement in disease-free survival in the recent series, even in the presence of a higher rate of positive margins.

Objective evaluation of efficacy is affected by the lack of prospective studies comparing local recurrence in comparison to, or in combination with, surgery. For radiotherapy, in the presence of microscopically positive margins, improved recurrence-free survival reported by UTMDACC [23] and Massachusetts General Hospital (MGH) [24] is counterbalanced by negative findings of MSKCC [17] and INT [16] studies. The indication for adjuvant radiotherapy for each patient of our series was evaluated within a framework of a multidisciplinary team comprising surgeons and radiotherapists. This resulted in a significantly higher probability for patients who underwent R1 surgery to receive postoperative radiotherapy (3 of 49 after R0 vs 4 of 13 after R1 surgery, Table 1).

The good results after R0 surgery in this series are to be considered in the light of the relatively high all-causes mortality that occurred during follow-up, despite the median age of the whole group of patients being 36 years. In particular, two patients died from causes directly related to the tumor treatment, and these data deserve careful consideration if we assume that even observation alone proved to be an effective option in high-risk patients [10,11].

## Conclusions

R0 surgery aided by IFS can decrease CR rate in patients with AF. For patients with tumors confined to the limbs, a particularly strict follow-up or non-surgical treatment could be appropriate because of the high risk of recurrence after R0 surgery in this subgroup. In selected cases, watchful waiting with close clinical follow-up could represent a viable strategy for limb tumors smaller than 5 cm [10,11]. Indeed, as the peculiar epidemiology of this tumor is unlikely to empower generous trial design, systematic risk weighting of individual components is, therefore, the most reasonable way to stratify patients and define a rationale for approaches other than surgery or in combination with it.

## Abbreviations

AF: Aggressive fibromatosis; AJCC: American Joint Committee on Cancer; APC: Adenomatous polyposis coli; CR: Cumulative recurrence; FAP: Familial adenomatous polyposis; IFS: Intraoperative frozen sections; INT: Istituto Nazionale dei Tumori; MGH: Massachusetts General Hospital; MRI: Magnetic resonance imaging; MSKCC: Memorial Sloan-Kettering Cancer Center; US: Ultrasound; UTMDACC: University of Texas MD Anderson Cancer Center.

## Competing interests

All of the authors of this manuscript declared that no financial or non-financial interests were related to this study or future application after publication.

## Authors’ contributions

EBe was responsible for manuscript writing and study design. AT, AC and RB gave comments and revised the manuscript. PM, EBo and BB were responsible for data acquisition and analysis. GV, GM and GC were in charge of correction of the manuscript, while TD, FV and BA collaborated on the whole project design. All authors read and approved the final manuscript.
